# Predictive validity of the braden scale for pressure injury risk assessment in adults: A systematic review and meta‐analysis

**DOI:** 10.1002/nop2.792

**Published:** 2021-02-25

**Authors:** Can Huang, Yuxia Ma, Chenxia Wang, Mengyao Jiang, Loretta Yuet Foon, Lin Lv, Lin Han

**Affiliations:** ^1^ Evidence‐Based Nursing Center School of Nursing Lanzhou University Lanzhou China; ^2^ Department of Endocrinology The First Hospital of Lanzhou University Lanzhou China; ^3^ The First Clinical Medical College Lanzhou University Lanzhou China; ^4^ Nursing Department Gansu Provincial Hospital Lanzhou China

**Keywords:** pressure ulcer, risk assessment, sensitivity and specificity, systematic review

## Abstract

**Aim:**

Pressure injuries are common adverse events in clinical practice, affecting the well‐being of patients and causing considerable financial burden to healthcare systems. It is therefore essential to use reliable assessment tools to identify pressure injuries for early prevention. The Braden Scale is a widely used tool to assess pressure injury risk, but the literature is currently lacking in determining its accuracy. This study aimed to evaluate the accuracy of the Braden Scale in assessing pressure injury risk.

**Design:**

Systematic review and meta‐analysis.

**Methods:**

Articles published between 1973–2020 from periodicals indexed in the PubMed, EMBASE, CINAHL, Web of Science and the Cochrane Library were selected. Two reviewers independently selected the relevant studies for inclusion. Data were analysed by the STATA 15.0 and the RevMan 5.3 software.

**Results:**

In total, 60 studies involving 49,326 individuals were eligible for this meta‐analysis. The pooled SEN, SPE, PLR, NLR, DOR and AUC were 0.78 (95% CI: 0.74 to 0.82), 0.72 (95% CI: 0.66 to 0.78), 2.80 (95% CI: 2.30 to 3.50), 0.30 (95% CI: 0.26 to 0.35), 9.00 (95% CI: 7.00 to 13.00) and 0.82 (95% CI: 0.79 to 0.85), respectively. Subgroup analyses indicated that the AUC was higher for prospective design (0.84, 95% CI: 0.81 to 0.87), mean age <60 years (0.87, 95% CI: 0.84 to 0.90), hospital (0.82, 95% CI: 0.79 to 0.86) and Caucasian population (0.86, 95% CI: 0.82 to 0.88). In addition, 18 was found to be the optimal cut‐off value.

**Conclusion:**

The evidence indicated that the Braden Scale had a moderate predictive validity. It was more suitable for mean age <60 years, hospitalized patients and the Caucasian population, and the cut‐off value of 18 might be used for the risk assessment of pressure injuries in clinical practice. However, due to the different cut‐off values used among included studies, the results had a significant heterogeneity. Future studies should explore the optimal cut‐off value in the same clinical environment.

## 1 INTRODUCTION

Pressure injuries (PIs), also known as decubitus ulcers, ischaemic ulcers, bedsores, pressure sores and pressure ulcers, are localized damage to the skin and underlying soft tissue usually over a bony prominence or related to a medical or other device (NPUAP, 2016). Individuals who are at high risk are those characterized by multiple risk factors that affect both the mechanical boundary conditions and the susceptibility and tolerance of the individual (National Pressure Ulcer Advisory Panel and Alliance, 2014). However, most PIs can be prevented if effective measures including systematic skin examination, risk assessment, bed and chair support surfaces, repositioning and mobilization, and nutritional support are implemented (Bredesen et al., 2015). Risk assessment is a central component of PI prevention (Coleman et al., 2013, National Pressure Ulcer Advisory Panel and Alliance, 2014), so it is important to use a valid and reliable assessment tool to identify high‐risk patients and implement appropriate interventions for the prevention of PIs.

Since the early 1960s, a variety of risk assessment tools have been developed with over 50 scales currently to determine the risk of PIs, such as the Norton Scale, the Waterlow Scale and the Braden Scale (Shi et al., 2019). The Braden Scale is the most common around the world due to its ease of use with wider risk factor incorporation (e.g. moisture and sensory perception) when compared to other scales (National Pressure Ulcer Advisory Panel and Alliance, 2014). However, it has been used in different population clinical settings, with a variety of re‐verification results. In order to take appropriate measures and prevent PI development early, practitioners must ascertain whether the Braden Scale can accurately identify the risk of PIs.

## 2 BACKGROUND

PIs are one of the most frequently occurring adverse events in hospitalized patients worldwide (Li et al., 2020, National Pressure Ulcer Advisory Panel and Alliance, 2014), which prolong hospital stay, increase medical expenses, decrease quality of life and result in increased nosocomial infection, disability, morbidity and mortality (Al Mutairi & Hendrie, 2018; Aloweni et al., 2019; Amir et al., 2017; Coleman et al., 2013; Ferris et al., 2019; Jackson et al., 2019; Mallow et al., 2013). The prevalence of PIs remains unacceptably high, ranging from 1.1%–26.7% in the hospital setting and 6%–29% in the community setting (Graves & Zheng, 2014). It has been estimated that the annual cost of treating PIs is $26.8 billion in the United States (Padula & Delarmente, 2019), €334.86 million to €2.59 billion in Europe (Severens et al., 2002) and A$983 million in Australia (Nguyen et al., 2015). A recent study noted that the cost of PI prevention was more cost‐effective than that of PI treatment across all clinical settings (Demarré et al., 2015). For these reasons, PI prevention is of great importance. An essential component of preventive strategies is the risk assessment of PI development in the individual.

Risk assessments tools are generally used to assess the risk of developing PIs, such as the Norton Scale, the Waterlow Scale and the Braden Scale. The ideal risk assessment tool must accurately identify individuals at risk, as well as those not at risk. The Norton Scale is the first structured risk assessment tool for predicting PIs, but it lacks the part of friction shear, which may result in the occurrence of PIs (National Pressure Ulcer Advisory Panel and Alliance, 2014). Although it was also developed to assess senile patients at risk of developing PIs, the Waterlow Scale cannot accurately identify those individuals who are not at risk, with the specificity of 32.9% (Serpa et al., 2009). The Baden Scale is based on six common risk factors including sensory function, moisture, activity, mobility, nutrition, shearing force and friction. A summative score reveals the level of risk where lower values are indicative of higher risk (Kelechi et al., 2013). Due to the ease of use and interpretation of the point system, the Braden Scale has quickly gained popularity among practitioners. However, in order to reflect the population characteristics and the medical culture of the country, the Braden Scale has been re‐verified by different researchers in the past 30 years. The sensitivity and specificity of it showed a wide range of differences from 50%–100% depending on the research subjects or conditions (Chou et al., 2013), and the cut‐off point differed as well (Cowan et al., 2012). Some studies (Chen et al., 2016; Pancorbo‐Hidalgo et al., 2010; Park et al., 2016) found that the Braden Scale offered the best balance between sensitivity and specificity. But a systematic review (Wei et al., 2012) revealed that the Braden Scale could not be used alone in assessing PIs’ risk in surgical patients. As a result, there is no consensus on predictive validity of the Braden Scale among different studies.

Given the importance of risk assessment for PI prevention, practitioners have used the Braden Scale in different population and clinical settings. However, it is unclear whether the Braden scale can accurately identify the risk of PIs in practice. The purpose of this study was to determine predictive validity of the Braden Scale and to explore the suitable population and optimal cut‐off value through a diagnostic method oriented meta‐analysis. Understanding the predictive validity, applicable population and optimal cut‐off value is beneficial for practitioners to identify the risk of PIs and take preventive measures early.

## 3 DESIGN

We conducted a systematic review and meta‐analysis. The study was performed in accordance with the guidelines from the Cochrane Handbook for Systematic Reviews of Diagnostic Test Accuracy from the Cochrane Collaboration (Macaskill et al., 2010) and Preferred Reporting Items for Systematic Review and Meta‐analysis (PRISMA) (Moher et al., 2009). Our study protocol was registered with the International Prospective Register of Systematic Reviews (PROSPERO) (CRD42020142181).

## 4 METHODS

### 4.1 Search strategy

The digital databases including PubMed, EMBASE, Web of Science, the Cochrane Library and the Cumulative Index of Nursing and Allied Health (CINAHL) were searched, from inception of each database to July 2020. In addition, we explored the bibliographies of relevant reviews in order to identify other potentially eligible studies. The literature search terms and strategies used are available in supplementary appendix 1.

### 4.2 Inclusion and exclusion criteria

The eligible studies must meet the following criteria: (a) patients were 18 years of age or older and had no PIs at time of admission; (b) the Braden Scale was used to identify the risk of PIs; (c) studies directly provided true positive (TP), false positive (FP), false negative (FN) and true negative (TN) for predicting PIs’ risk or with data available regarding these statistics; (d) the definition and classification of PIs were produced by one of the accepted standards, such as the National Pressure Ulcer Advisory Panel (NPUAP), the European Pressure Ulcer Advisory Panel (EPUAP), the Agency for Health Care Policy and Research (AHCPR), the International Classification of Diseases, Ninth Revision (ICD‐9), and the Bergstrom and others; and (e) the cross‐sectional study and the cohort study were included.

Exclusion criteria were as follows: (a) studies failed to obtain a complete data; (b) letter, comment and meeting abstract; and (c) duplicate publications.

### 4.3 Study selection

Two reviewers independently screened titles and abstracts for eligibility with the consistent accomplishment of a pilot literature selection. The full text was read if the abstract and title cannot be determined for inclusion. In case of disagreement, a third reviewer resolved the conflict between them.

### 4.4 Data extraction

Two reviewers extracted data into a spreadsheet independently and resolved any discrepancies through discussion to reach a consensus. For each study included, the following information was extracted: first author, publication year, country, study design, age, gender, sample size, cut‐off value, reference standard, TP, FP, TN and FN.

### 4.5 Quality assessment

The Quality Assessment of Diagnostic Accuracy Studies Ⅱ (QUADAS‐Ⅱ) (Whiting et al., 2011) was used to assess the quality of each of the included studies. It contains four domains: patient selection, index test, reference standard, and flow and timing, classifying the methodological quality as having a low, high or unclear risk of bias. Two reviewers independently rated the applicability and risk of bias, and any conflict was resolved by a third reviewer.

### 4.6 Statistical analysis

All statistical analyses were performed using STATA 15.0 (Stata, College Station, TX, USA) and Review Manager 5.3 software (Cochrane Collaboration, Oxford, UK). The bivariate meta‐analysis model was selected to calculate the pooled sensitivity (SEN), specificity (SPE), positive likelihood ratio (PLR), negative likelihood ratio (NLR), diagnostic odds ratio (DOR) and their corresponding 95% confidence intervals (95% CIs) (Reitsma et al., 2005). Furthermore, the summary receiver operator characteristic (SROC) curve was constructed and the area under the curve (AUC) was calculated to quantify the diagnostic power (Jones & Athanasiou, 2005). With respect to the value, a value of 0.5 was deemed informative, 0.5 < AUC≤0.7 was considered less accurate, 0.7 < AUC≤0.9 was thought to be moderate, 0.9 < AUC<1 was deemed very accurate, and AUC = 1 was considered a perfect test (Greiner et al., 2000). Heterogeneity was analysed by *I^2^* statistics. ≤25%, 25%<*I^2^* ≤ 75% and > 75% indicated respectively low, moderate and high heterogeneity between studies (Higgins et al., 2003). Subgroup analysis and sensitivity analysis were used to identify the sources of heterogeneity. Subgroup analysis was performed under the following covariates: (a) study design (prospective vs. retrospective); (b) mean age (<60 years vs. ≥60 years); (c) setting (hospital vs. non‐hospital); (d) ethnicity (Asian population vs. Caucasian population); and (e) reference standard (authoritative vs. non‐authoritative). In addition, we used Deeks’ funnel plot to assess any potential publication bias (Deeks et al., 2005).

## 5 RESULTS

### 5.1 Search results

**FIGURE 1:**
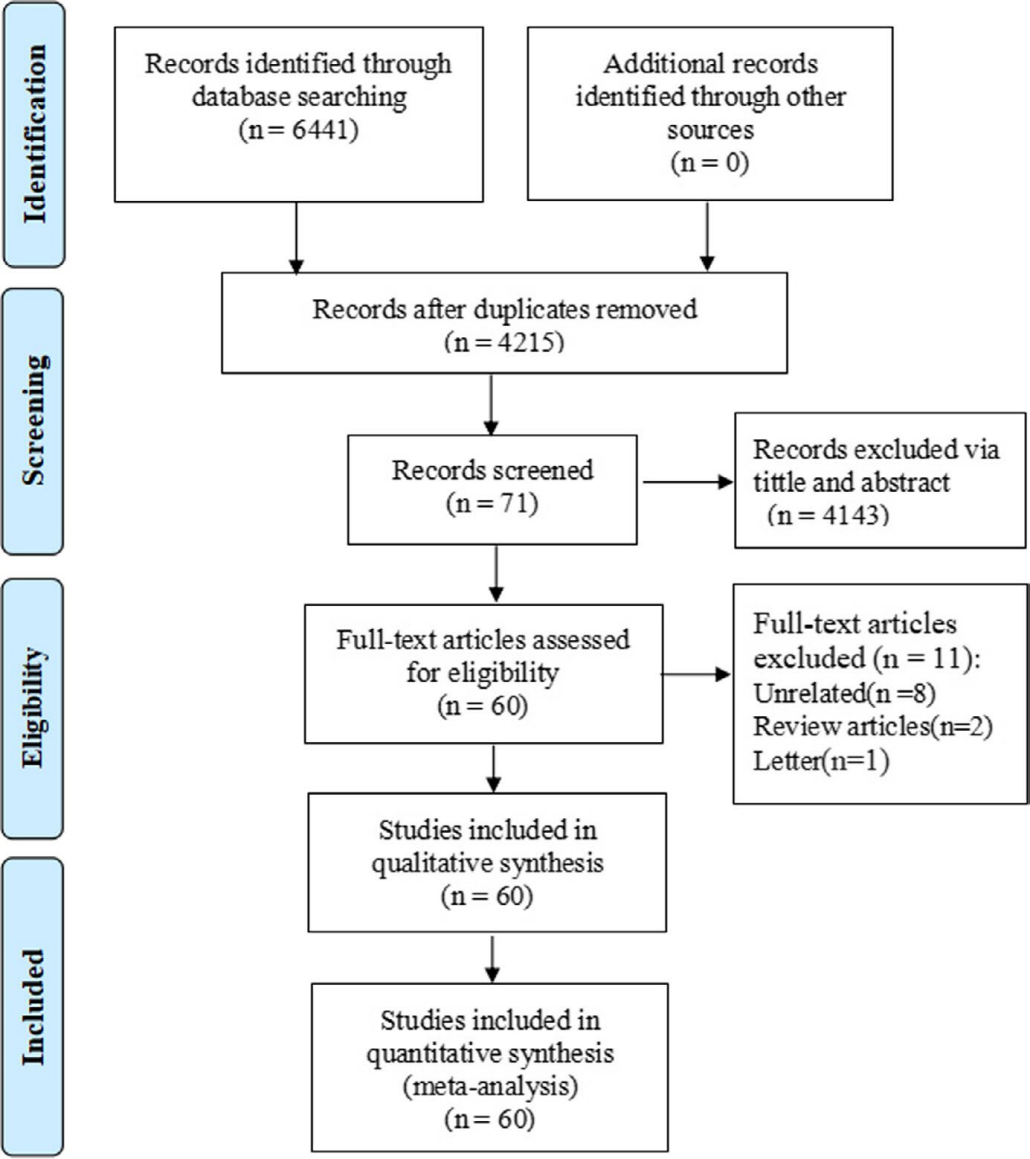
Flow diagram of article of selection

A total of 6,441 publications were identified in our initial search. 4,215 studies remained after removing duplications. After scanning titles and abstracts, 71 studies were identified for further examination. By reviewing the full text of the remaining articles, 11 studies with insufficient data or no relevance to the diagnosis were rejected. Finally, a total of 60 studies were included in this review (Table 1). The detailed screening process is presented in Figure 1.

**TABLE 1:** Characteristics of the included studies

Author/year	Country	Study design	Setting	Age (year)	Gender (female/male)	Sample size	Cut‐off	TP	FP	FN	TN
Lim et al., (2019)	Singapore	R	Ward	68 ± 17.1	80/119	199	≤17	68	25	32	74
Limaserrano et al., (2018)	Spain	P	ICU	63.74 ± 16.12	129/206	335	≤12	21	82	6	226
Han et al., (2018)	Korea	R	ICU	62.37 ± 14.32	223/377	600	≤16	242	131	58	169
Chen et al., (2017)	China	R	Ward	60.5 ± 15.6	962/1563	2,525	≤14	63	727	13	1722
Deng et al., (2017)	China	R	ICU	58 ± 17	119/349	468	≤16	70	80	24	294
Roca‐Biosca et al., (2017)	Spain	P	ICU	59.34–62.92	NR	295	≤12	34	168	7	86
Šáteková et al., (2017)	Czech Republic	P	LTCF	73.89 ± 10.12	NR	100	≤15	12	40	2	46
Roa Díaz et al., (2017)	Colombia	P	Ward	≥18	407/531	938	≤18	43	361	5	529
Griswold et al., (2017)	USA	R	ICU	48.3 ± 18.2	743/1917	2,660	≤12	50	1,299	11	1,299
Jin et al., (2015)	Korea	R	ICU	62.66 ± 17.98	NR	965	≤18	615	87	92	171
Kumari et al., (2015)	Indian	P	Ward	≥18	NR	100	≤17	23	0	8	69
García‐Díaz et al., (2015)	Spain	P	LTCF	82.3 ± 10.07	99/254	353	≤15	34	85	6	200
Freitas and Alberti, (2013)	Brazil	P	LTCF	82.5 ± 12.1	126/57	183	≤18	37	56	0	90
Hyun et al., (2013)	USA	R	ICU	58.7 ± 15.2	3317/4473	7,790	≤13	5,901	124	1655	110
Cowan et al., (2012)	USA	R	ACU	71.0 ± 10.6	7/206	213	≤18	65	34	35	79
Fromantin et al., (2011)	France	P	Ward	16–89	434/148	582	≤18	26	100	3	453
Costa and Caliri, (2011)	Brazil	P	ICU	≥18	NR	53	≤14	19	18	1	15
Serpa et al., (2011)	Brazil	R	ICU	60.9 ± 16.5	24/48	72	≤13	6	11	2	53
Cho and Noh, (2010)	Korea	R	ICU	62.33 ± 15.5	282/433	715	≤13	32	355	10	318
de Souza et al., (2010)	Brazil	P	LTCF	76.6 ± 9.2	129/104	233	≤17	27	48	10	148
Feuchtinger et al., (2010)	Germany	P	ICU	62.0 ± 12.1	22/31	53	≤16	20	19	6	8
Chan et al., (2009)	China	P	ACU	82.2 ± 7.35	167/30	197	≤16	12	64	6	115
Kim et al., (2009)	Korea	P	ICU	58.1 ± 1.2	74/145	219	≤14	37	54	3	125
Tannen et al., (2008)	Netherlands	P	LTCF	81.2 ± 10.2	7301/2797	10,098	≤18	2,337	3,591	834	3,336
Oh et al., (2007)	Korea	P	Ward	51.1	885/997	1882	≤18	4	114	0	1764
Lahmann et al., (2006)	Germany	P	LTCF	81.9 ± 12.2	3843/1003	4,846	≤20	406	2,507	268	1665
Tannen et al., (2006)	Germany	P	LTCF	84.6 ± 8.0	2873/626	3,499	≤20	243	1853	25	1,378
Sanada et al., (2006)	Indonesia	P	ICU	50.9 ± 17.0	33/72	105	≤12	28	32	7	38
Lahmann et al., (2005)	Germany	P	LTCF	83.6	NR	1,347	≤20	149	712	8	458
Defloor and Grypdonck, (2005)	Belgium	P	LTCF	84.6 ± 7.9	1403/369	1772	≤16	160	724	27	861
Jalali and Rezaie, (2005)	Iran	P	Ward	60	130/100	230	≤16	39	0	35	156
Kwong et al., (2005)	China	P	ACU	54.1 ± 16.9	176/253	429	≤14	8	118	1	302
Seongsook et al., (2004)	Korea	P	ICU	62	48/64	112	≤16	34	57	1	20
Cho et al., (2004)	Korea	P	Ward	55.3 ± 15.8	1555/2339	3,894	≤16	94	248	15	3,524
Marrie et al., (2003)	Canada	R	Ward	61.0 ± 18.0	90/98	188	≤16	35	33	11	109
Lee, (2003)	Korea	P	ICU	54.1	18/48	66	≤16	26	14	4	22
Bergstrom and Braden, (2002)	USA	P	LTCF	19–99	NR	825	≤18	74	165	32	554
Schoonhoven et al., (2002)	Netherlands	P	Ward	67.2 ± 14.8	NR	2,190	≤18	105	628	136	1,321
Bergquist and Frantz, (2001)	USA	R	LTCF	78.78 ± 8.38	1070/641	1696	≤19	75	604	32	985
Halfens et al., (2000)	Netherlands	P	Ward	60.9 ± 18.3	153/167	320	≤20	34	82	13	191
Lewicki et al., (2000)	USA	P	Ward	62 ± 11.59	83/254	337	≤14	9	26	7	295
Hagisawa and Barbenel, (1999)	Japan	P	Ward	NR	NR	275	≤16	5	0	7	263
Bergstrom et al., (1998)	USA	P	LTCF	63 ± 16	94/161	255	≤18	49	52	12	142
Goodridge et al., (1998)	Canada	P	Ward	78.6 ± 8.5	NR	330	≤19	22	134	10	164
Lyder et al., (1998)	USA	P	Ward	71.0 ± 6.5	21/15	36	≤16	5	0	9	22
Schue and Langemo, (1998)	USA	R	Ward	69.2 ± 10.9	0/170	170	≤18	33	50	13	74
Pang and Wong, (1998)	China	P	Ward	45–92	54/52	106	≤18	19	32	2	53
Baldwin and Ziegler, (1998)	USA	P	ICU	31.7 ± 10.9	20/16	36	≤10	10	1	1	24
Olson et al., (1998)	Canada	P	Ward	54.8–62.4	NR	128	≤16	9	19	2	98
Watkinson, (1997)	UK	P	Ward	82.7	68/24	92	≤16	14	18	1	59
Capobianco and McDonald, (1996)	USA	P	Ward	66.9 ± 19.3	32/18	50	≤18	10	6	4	30
Harrison et al., (1996)	Canada	P	ACU	60.0 ± 19.0	362/376	738	≤19	147	176	72	343
VandenBosch et al., (1996)	USA	P	Ward	67 ± 13.8	54/49	103	≤17	17	30	12	44
Ramundo, (1995)	USA	P	LTCF	NR	NR	48	≤18	7	27	0	14
Braden and Bergstrom, (1994)	USA	P	LTCF	75.9 ± 9.45	73/29	102	≤18	22	19	6	55
Barnes and Payton, (1993)	USA	P	AVU	50–90	178/183	361	≤16	16	32	6	307
Salvadalena et al., (1992)	USA	P	Ward	72.0 ± 13.0	63/34	99	≤16	8	24	12	55
Choi and Song, (1991)	Korea	P	Ward	54.1	57/89	146	≤16	13	8	3	122
Langemo et al., (1991)	USA	P	LTCF	66	NR	25	≤18	4	7	3	11
Bergstrom et al., (1987)	USA	P	ICU	58.5 ± 14.5	32/28	60	≤16	20	13	4	23

Note

Abbreviations: ACU, acute care unit; ICU, intensive care unit; LTCF, long‐term care facility; TP, true positive; FP, false positive; FN, false negative; TN, true negative; R, retrospective; P, prospective; and NR, no report.

### 5.2 Study characteristics

The baseline characteristics of these included studies are shown in Table 1. In total, 49,326 individuals were involved in this meta‐analysis, whose mean age ranged from 31–84 years. These studies were published between 1987–2019. 45 studies were performed in hospitals and 15 in long‐term care facilities (LTCF). Of all studies, 47 were prospective and 13 were retrospective in nature. Among these studies, 41 studies were performed in Caucasian populations, while 19 studies were conducted in Asian populations. The cut‐off point showed a wide range between 10–20 out of the total score of 23.

### 5.3 Results of risk of bias

**FIGURE 2:**
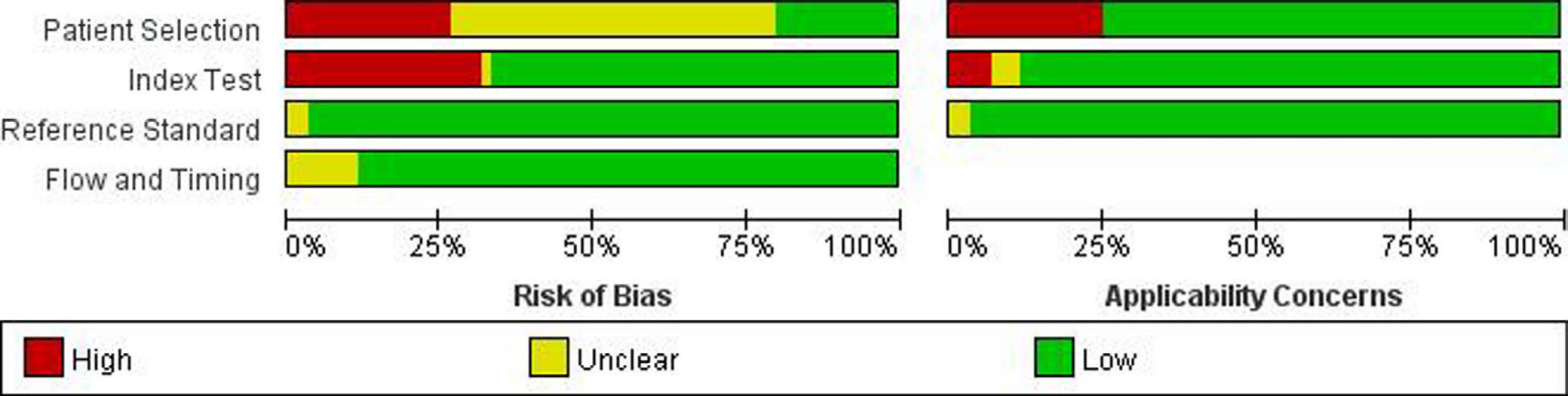
Study quality assessment results

The risk of bias and applicability were assessed. In the risk of bias, a low risk of patient selection was shown in 11 (18%) studies, and 39 (65%) studies were observed to have low risk in terms of the index test. Reference standard in 58 (97%) studies were judged to have a low risk of bias, and 50 (83%) studies belonged to low risk in the domain of flow and timing. In applicability, 44 (73%) studies were deemed to be low risk in the patient selection, 51 (85%) studies in the index test and 58 (97%) studies in the reference standard. Details regarding risk of bias and applicability are summarized in Figure 2.

### 5.4 Predictive validity of the Braden Scale

**FIGURE 3:**
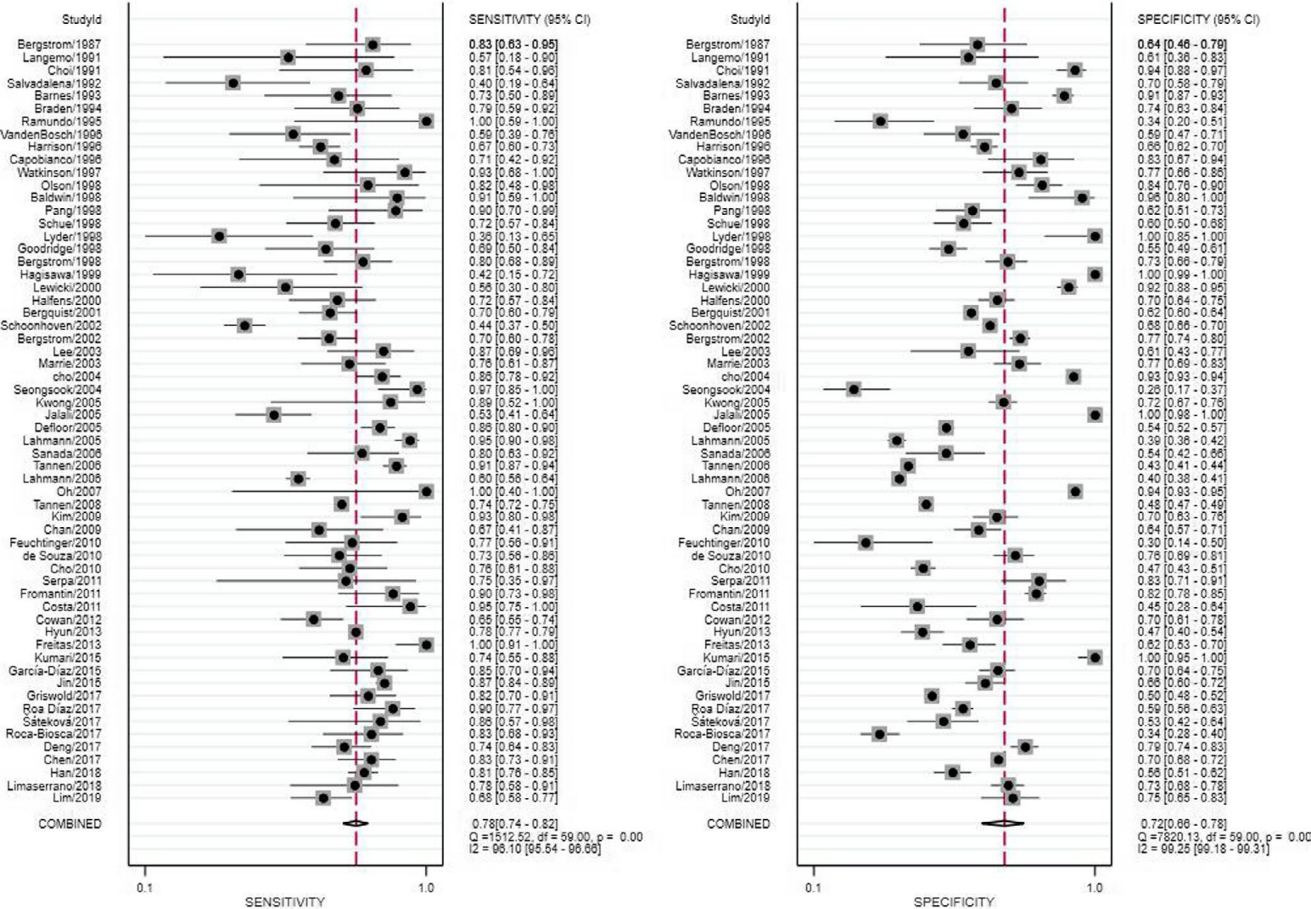
Sensitivity and specificity of included studies

**FIGURE 4:**
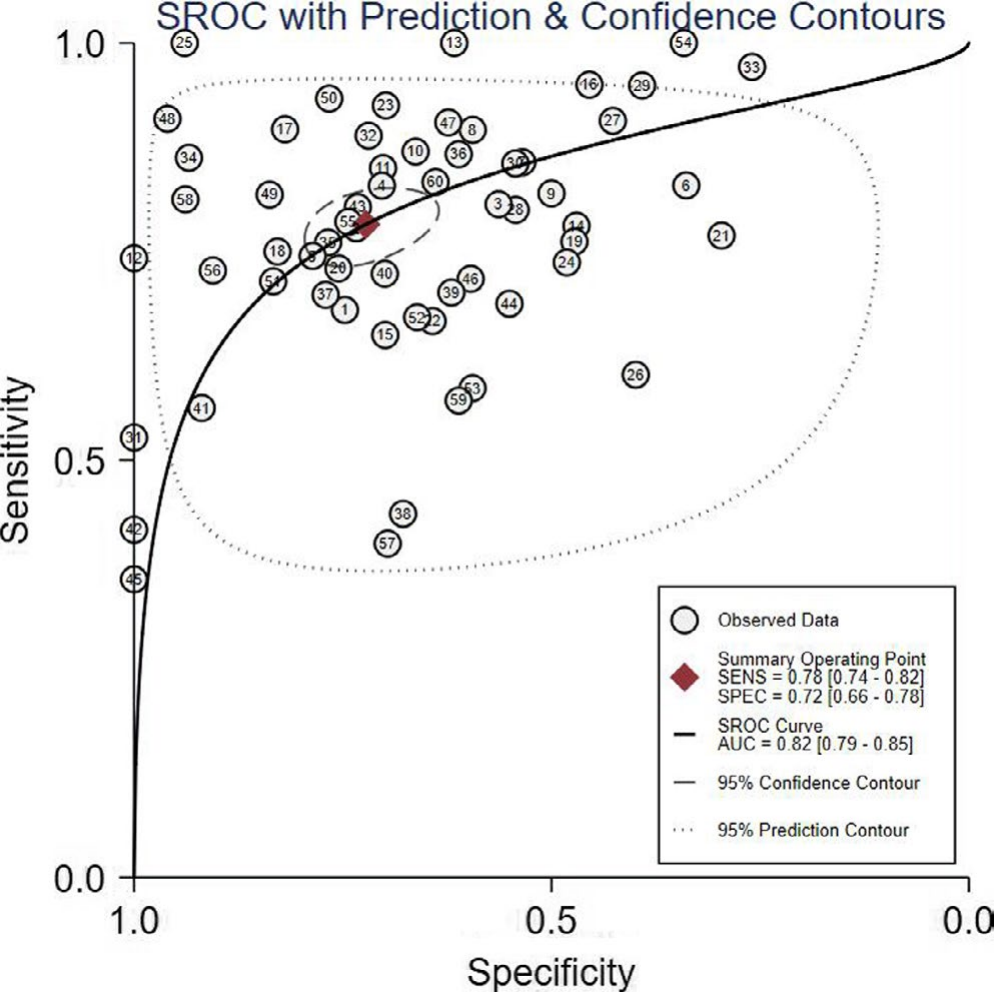
Summary receiver operating characteristic curve

The pooled SEN was 0.78 (95% CI: 0.74 to 0.82), and the pooled SPE was 0.72 (95% CI: 0.66 to 0.78) (Figure 3). The pooled PLR and NLR were 2.80 (95% CI: 2.30 to 3.50) and 0.30 (95% CI: 0.26 to 0.35), respectively, which yielded a DOR of 9.00 (95% CI: 7.00 to 13.00). In addition, the SROC AUC was 0.82 (95% CI: 0.79 to 0.85) (Figure 4). *I^2^* values in SEN and SPE reached 96.10% (*c^2^* = 1512.52, *p* <.05) and 99.17% (*c^2^* = 7820.13, *p* <.05), respectively.

### 5.5 Threshold effect

Visual inspection of forest plots and SROC curves, as well as Spearman's correlation of 0.334 (*p* =.009), suggested the presence of a threshold effect to some extent. The pooled results of different cut‐off points are shown in Table 2.

**TABLE 2:** Summary results of meta‐analysis

	*n*	SEN (95% CI)	SPE (95% CI)	PLR (95% CI)	NLR (95% CI)	DOR (95% CI)	AUC (95% CI)
Total	60	0.78 (0.74–0.82)	0.72 (0.66–0.78)	2.80 (2.30–3.50)	0.30(0.26–0.35)	9.00 (7.00–13.00)	0.82 (0.79–0.85)
Outlier excluded	52	0.79 (0.76–0.82)	0.70 (0.64–0.74)	2.60 (2.20–3.10)	0.30 (0.26–0.35)	8.00 (6.00–11.00)	0.82 (0.78–0.85)
Study design							
Prospective	47	0.80 (0.74–0.84)	0.75 (0.67–0.82)	3.20 (2.40–4.30)	0.27 (0.22–0.33)	12.00 (8.00–18.00)	0.84 (0.81–0.87)
Retrospective	13	0.77 (0.73–0.81)	0.65 (0.58–0.71)	2.20 (1.80–2.60)	0.36 (0.30–0.43)	6.00 (5.00–9.00)	0.78 (0.75–0.82)
Mean age							
<60	16	0.83 (0.79–0.87)	0.78 (0.68–0.85)	3.70 (2.50–5.50)	0.22 (0.16–0.28)	17.00 (10.00–31.00)	0.87 (0.84–0.90
≥60	44	0.77 (0.71–0.81)	0.71 (0.62–0.79)	2.70 (2.10–3.40)	0.33 (0.28–0.39)	8.00 (6.00–11.00)	0.81 (0.77–0.84)
Setting							
Hospital	46	0.76 (0.72–0.80)	0.77 (0.69–0.84)	3.40 (2.50–4.50)	0.31 (0.26–0.36)	11.00 (7.00–16.00)	0.82 (0.79–0.86)
ACU	5	0.68 (0.62–0.74)	0.74 (0.63–0.83)	2.60 (1.70–4.10)	0.43 (0.33–0.56)	6.00 (3.00–12.00)	0.72 (0.67–0.75)
ICU	17	0.83 (0.79–0.86)	0.59(0.49–0.68)	2.00 (1.60–2.50)	0.29(0.23–0.37)	7.00 (4.00–10.00)	0.83 (0.79–0.86)
Wards	24	0.71 (0.64–0.78)	0.87 (0.78–0.93)	5.60 (3.20–9.80)	0.33 (0.26–0.42)	17.00 (9.00–33.00)	0.83 (0.80–0.86)
LTCF	14	0.84 (0.77–0.90)	0.58 (0.51–0.66)	2.00 (1.70–2.40)	0.27 (0.18–0.39)	8.00 (5.00–12.00)	0.77 (0.74–0.81)
Ethnicity							
Asian	19	0.80 (0.74–0.85)	0.84 (0.67–0.93)	4.90 (2.40–10.10)	0.24 (0.19–0.30)	20.00 (10.00–43.00)	0.82 (0.79–0.86)
Caucasian	41	0.77 (0.72–0.82)	0.68 (0.61–0.73)	2.40 (2.00–2.80)	0.33 (0.27–0.40)	7.00 (5.00–10.00)	0.86 (0.82–0.88)
Cut‐off							
≤15	15	0.79 (0.76–0.82)	0.66 (0.55–0.75)	2.30 (1.70–3.20)	0.31 (0.25–0.40)	7.00 (4.00–12.00)	0.80 (0.76–0.83)
16	19	0.75 (0.67–0.82)	0.85 (0.70–0.93)	5.00 (2.50–10.20)	0.29 (0.23–0.37)	17.00 (8.00–36.00)	0.84 (0.80–0.87)
17	4	0.69 (0.61–0.76)	0.86 (0.50–0.97)	4.90 (1.00–25.00)	0.36 (0.23–0.55)	14.00 (2.00–103.00)	0.73 (0.69–0.77)
18	15	0.82 (0.73–0.89)	0.70 (0.62–0.77)	2.70 (2.10–3.60)	0.25 (0.16–0.39)	11.00 (6.00–20.00)	0.83 (0.79–0.86)
≥19	7	0.78 (0.65–0.87)	0.54 (0.44–0.63)	1.70 (1.40–2.00)	0.41 (0.26–0.65)	4.00 (2.00–7.00)	0.67 (0.63–0.71)

Note

Abbreviations: SEN, sensitivity; SPE, specificity; PLR, positive likelihood ratio; NLR, negative likelihood ratio; DOR, diagnostic odds ratio; AUC, area under the curve; 95% CIs, 95% confidence intervals; ACU, acute care unit; ICU, intensive care unit; LTCF, long‐term care facility.

### 5.6 Subgroup analyses

In order to explore possible heterogeneity factors, we performed subgroup analyses based on study design (prospective vs. retrospective), mean age (<60 years vs. ≥60 years) (Matsumoto et al., 2018), setting (hospital vs. LTCF) and ethnicity (Asian population vs. Caucasian population). The pooled diagnostic parameters for subgroup analyses are summarized in Table 2.

### 5.7 Sensitivity analysis and publication bias

**FIGURE 5:**
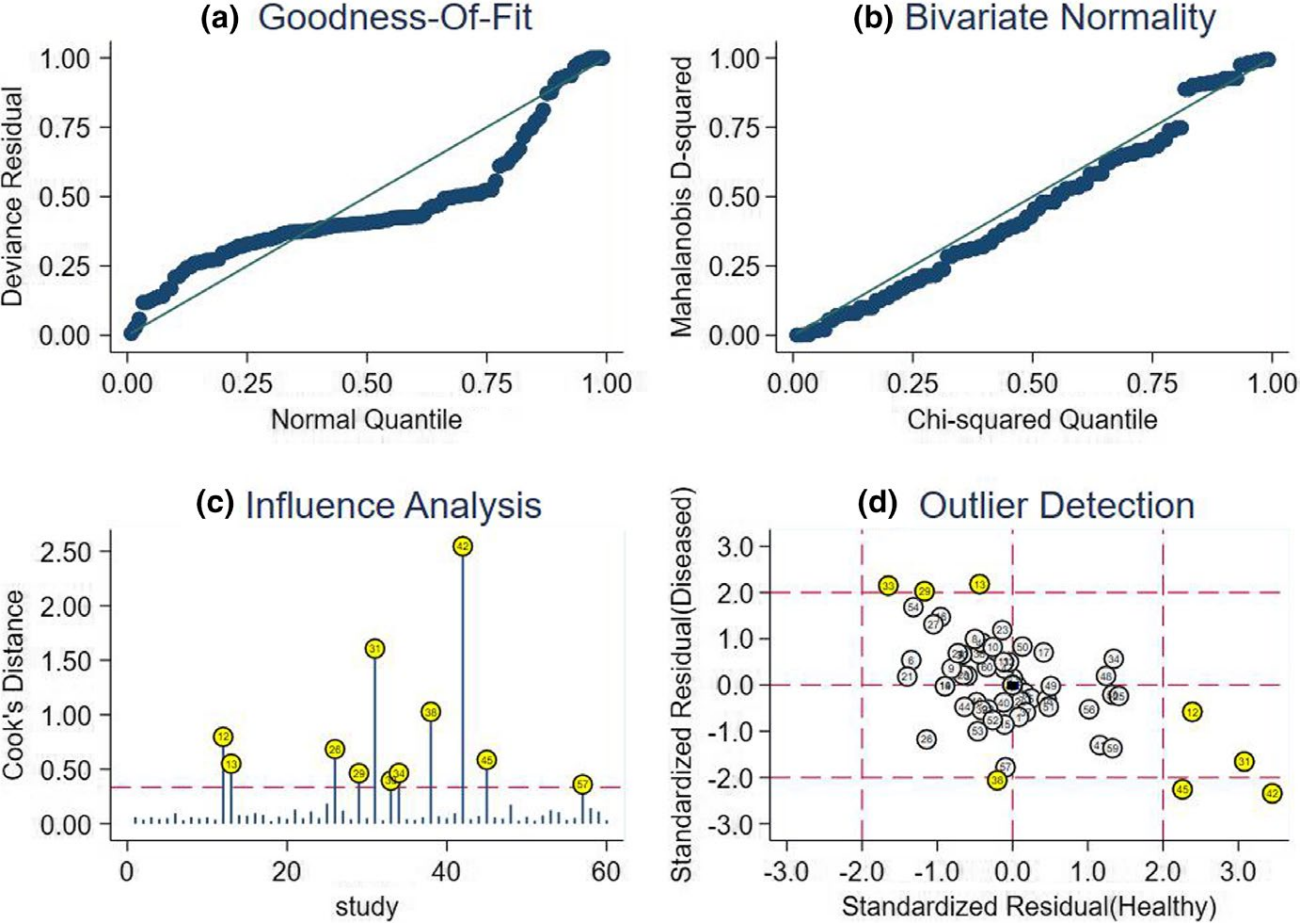
Sensitivity analysis results. a Goodness of fit; b bivariate normality; c influence analysis; and d outlier detection

**FIGURE 6:**
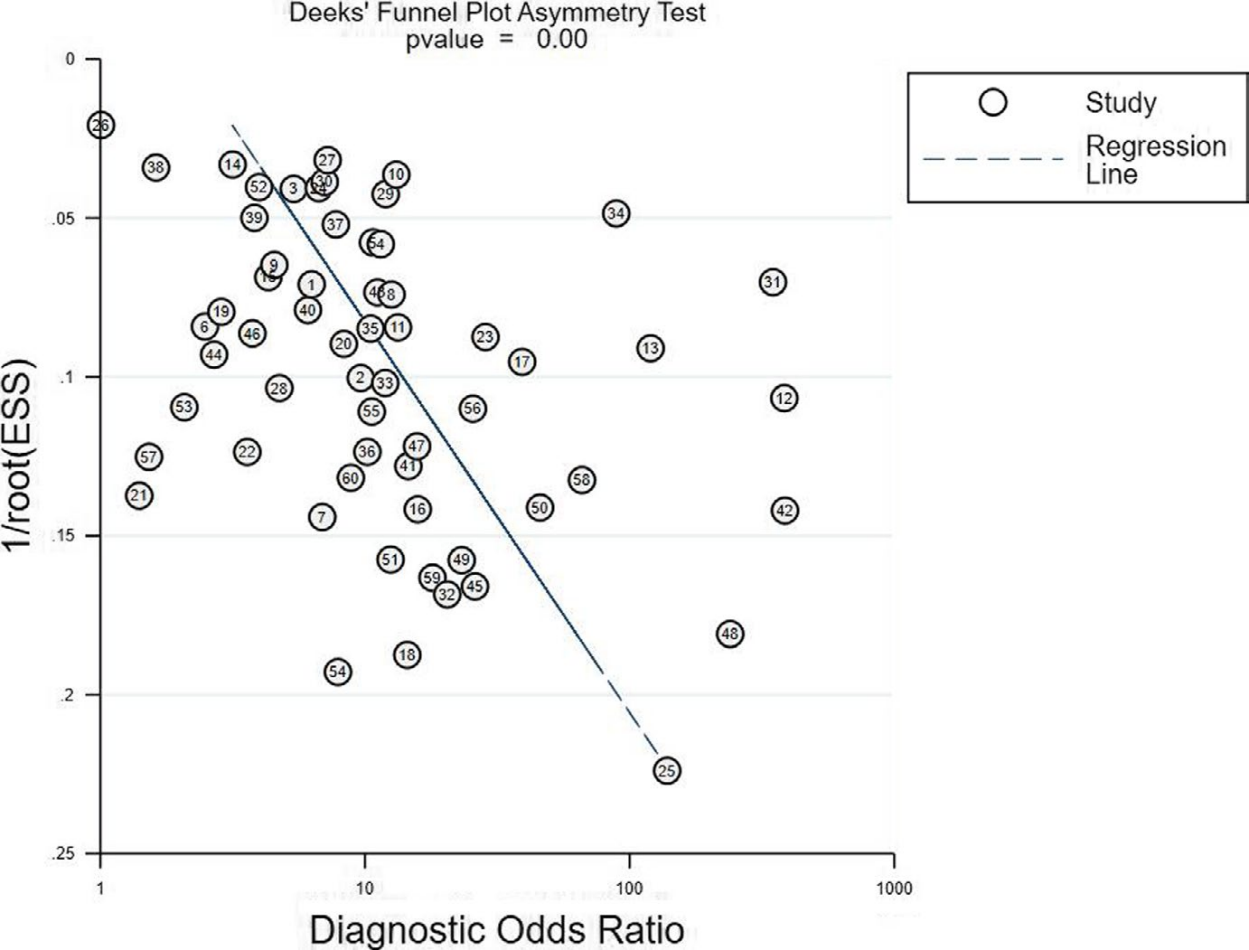
Deek's funnel plot asymmetry test for identifying publication bias

We carried out sensitivity analysis to assess the result reliability in Figure 5. The goodness of fit and bivariate normality showed that the included studies had only minimal influence on the overall estimates. Influence analysis and outlier detection identified eight outlier studies. After excluding these outlier studies, the SEN increased from 0.78–0.79, the SPE dropped from 0.72–0.70, the PLR decreased from 2.80–2.60, the NLR showed no change from 0.30–0.30, the DOR decreased from 9.00–8.00, and the AUC showed no change from 0.82–0.82, which suggested that the random‐effects bivariate model was robust for the calculation of the pooled estimates. Finally, Deeks’ funnel plot asymmetry test was used to assess the potential publication bias. The funnel plot (Figure 6) was not fully symmetrical, suggesting publication bias may exist in this meta‐analysis (*p* <.05).

## 6 DISCUSSION

This meta‐analysis included 60 studies involving 49,326 patients. The pooled SEN, SPE, PLR, NLR, DOR and AUC were 0.78, 0.72, 2.80, 0.30, 9.00 and 0.82, respectively. Subgroup analyses indicated that the Braden Scale was more accurate in assessing the risk of PIs for mean age <60 years, hospitalized patients and Caucasian population. When the cut‐off value was 18, the Braden Scale was the most effective in identifying PIs’ risk.

The results indicated that the probability of a positive result was 78% when the Braden Scale was used to assess a person who actually developed PI, and the probability of a negative result was 72% when the Braden Scale was applied to assess a person who did not actually develop PI. The pooled PLR and NLR were also calculated in order to evaluate the diagnostic accuracy in clinical level. The pooled PLR value was 2.80, suggesting that the probability of PI in a person with a positive test was 2.80‐fold higher than that in a healthy individual. By contrast, the pooled NLR indicated that the probability of not having PI in a person with a negative test was 30%. Meanwhile, DOR demonstrated a high level of overall accuracy. DOR, which is found by dividing PLR by NLR, can range from 0–infinity, and a higher DOR represents higher accuracy (Deeks, 2001). Finally, the AUC (0.83) showed that the Braden Scale had a moderate predictive validity for PI risk assessment. In addition, compared with the Waterlow Scale (0.75) and the Norton Scale (0.55) (Park & Lee, 2016), the Braden Scale had a higher SEN. Based on the results above, it was suggested that the Braden Scale might be more suitable for PI risk assessment. The reasons are shown as follows: (a) a good assessment tool was high in both SEN (true‐positive rate) and SPE (true‐negative rate), which was generally unavailable in clinical settings (Park et al., 2015). PI risk assessment was a screening inspection that preferred a higher sensitive tool rather than a higher specific tool. When the AUC was the same, the higher SEN was better in identifying the risk of PIs, which was beneficial to taking PI preventive interventions in time; and (b) risk assessment tools of PIs were based on its risk factors. The Braden Scale included more factors than the Waterlow Scale and the Norton Scale, such as restricted mobility, limited sensory perception and excess moisture. They are important factors that lead to the development of PIs. Considering that preventive measures are more cost‐effective than therapeutic measures for PIs (Zarei et al., 2019), it is suggested that nursing staffs apply the Braden Scale to identify factors that impact on an individual's risk in clinical practice.

Given the significant heterogeneity among included studies, we carried out threshold analyses. Spearman's correlation of 0.334 (*p* =.009) suggested the presence of a threshold effect to some extent. Threshold effect occurs when different cut‐off values are used to define a positive test result in different studies, affecting the reported sensitivity and specificity of the test (Mahmood et al., 2019). In this meta‐analysis, the cut‐off value ranged from 10–20, which indicated that the cut‐off value might be the primary reason of significant heterogeneity. Moreover, we conducted cut‐off‐stratified analyses according to the values ≤15, 16, 17, 18 and ≥19. Compared with other cut‐off values, 16 and 18 were better in SEN (0.75 and 0.82), SPE (0.85 and 0.70) and AUC (0.84 and 0.83), which were also widely used in clinical practice nowadays. As a result, it seemed that the cut‐off value of 18 might be the best choice. The possible reason was that a risk assessment tool for PIs was not a diagnostic tool for the incidence of PIs but instead a screening tool assessing the risk of PIs. The cut‐off value of 18 had a higher SEN than that of 16. However, in view of the characteristics in the specific clinical setting, whether the value of 18 can be treated as the optimal cut‐off was unknown. Future studies could explore this issue among different populations, such as medical, surgical, critical and elderly patients. In addition, it is necessary to conduct multi‐centre, large‐sample studies in order to verify the effectiveness of 16 and 18 in PI risk assessment.

Based on the subgroup analyses, we found that results showed a higher level of accuracy among prospective studies (AUC: 0.84) than retrospective design (AUC: 0.78), which may be attributed to more rigorous design in the prospective studies. Although there was no significant difference in the AUC (0.87 vs. 0.81) between the young and middle‐aged population and the elderly, the pooled SEN and SPE of the young and middle‐aged population were 0.83 and 0.78, while those of the elderly were 0.77 and 071. Based on these, we found that the Braden Scale was more accurate in the young and middle‐aged population than in elderly. The possible reason was that older people developed chronic diseases due to their declined physiological reserve (Jaul, 2010), which was not considered in the Braden Scale. Moreover, oxygenation and perfusion situations that do not exist in the Braden Scale may also affect PI development in elderly people (Iranmanesh et al., 2012). An additional finding was that the Braden Scale had a higher diagnostic accuracy in the hospital than in the LTCF (AUC, 0.82 vs. 0.77). Such result correlated with some published studies (Park et al., 2015; Wei et al., 2020), but different from another study (Wei et al., 2012). The small number of selected studies might contribute to the difference. Further studies with an increased number of studies could clarify the inconsistency issue among studies. Moreover, compared with its use in the acute care unit (AUC: 0.72, SEN: 0.68, SPE: 0.74) and intensive care unit (AUC: 0.83, SEN: 0.83, SPE: 0.59), the Braden scale was more suitable for use in the general wards (AUC: 0.83, SEN: 0.71, SPE: 0.87). The reason was that some other risk factors for PIs in the acute care unit and intensive care unit including emergency environment, sedation, vasoactive agents, mechanical ventilation, incontinence and oedema were not found in the Braden Scale. In terms of ethnicity, the Braden Scale demonstrated higher diagnostic accuracy in the Caucasian population than in Asian population (AUC, 0.86 vs. 0.82). Taking cultural difference into account, the Braden Scale, which was developed in the United States, might be more suitable to Caucasian population.

### 6.1 Strengths and limitations

The strengths of this meta‐analysis included the large number of patients retained in the quantitative synthesis. Furthermore, this is the first meta‐analysis on the overall accuracy of the Braden Scale for identifying PI risk. In addition, we performed threshold analyses and cut‐off‐stratified analyses, and identified the optimal cut‐off value, which played an important role in determining the risk of PIs. More importantly, sensitivity analysis was performed in order to find outlier studies. After removing the outliers and performing the same analyses for the remaining studies, we found that the overall parameters of diagnostic accuracy did not change significantly, which suggested that the random‐effects bivariate model was robust for the calculation of the pooled estimates. However, there are still several limitations. First, we have implemented a comprehensive systematic literature review, yet the language of the included studies was limited to English and Chinese, which might lead to publication bias. Second, because the cut‐off value of 16 was only found in the 4 original studies, the pooled parameters in the systematic review had a limited interpretation. Future studies should conduct more original researches to compare the effectiveness of 18 with that of 17.

### 6.2 Implication for practice

The Braden Scale is more suitable to identify the risk of PI for mean age <60 years, hospitalized patients and the Caucasian population. It appears that 18 is the optimal cut‐off value in clinical practice.

## 7 CONCLUSION

The Braden Scale has a moderate predictive validity for PI risk assessment, and it is more suitable for mean age <60 years, hospitalized patients and Caucasian population, compared with mean age ≥60 years, long‐term care facility and Asian population. Meanwhile, the cut‐off value of 18 afforded the best choice in SEN and AUC, and could be recommended for use in clinical practice. Future studies should explore the optimal cut‐off in the specific environments.

## CONFLICT OF INTEREST

The authors declare no conflict of interest.

## AUTHOR CONTRIBUTIONS

C.H., Y.M., L.H.: Study design. C.H., Y.M., M.J.: Data collection. C.H., Y.M., C.M.: Data analysis. C.H., Y.M., M.J., C.W., L.H.: Manuscript writing and revisions for important intellectual content.

## Supporting information

Supplementary Material

## Data Availability

The data used to support the findings of this study are available from the corresponding author on reasonable request.
