# Supplementary material for: Predictive validity of the braden scale for pressure injury risk assessment in adults: A systematic review and meta‐analysis
Source: Nurs Open. 2021 Feb 25;8(5):2194–207. doi: 10.1002/nop2.792 (PMC8363405; doi:10.1002/nop2.792)
Supplement: Supplementary file 1 — Supplementary Material [file NOP2-8-2194-s001.docx]

**Web of science：**

#1 TS:pressure ulcer* OR pressure injur* OR pressure sore* OR pressure dama* OR decubitus ulcer*OR decubitus sore* OR Bedsore* OR bed sore*)

#2 TS: (braden near (score* or scale* or tool* or assess*)) *OR* TS: (risk near/2 assess*) *OR* TS: ((assess* or predict*) next (tool* or score* or scale* or instrument* or equipment* or device*))

#3 #2 AND #1

**Cochrane：**

#1 MeSH descriptor: [Pressure Ulcer] explode all trees

#2 (decubitus next (ulcer* or sore*)):ti,ab,kw OR (bedsore* or (bed next/1 sore*)):ti,ab,kw OR (pressure* next (wound* or sore* or ulcer* or injur* or damag*)):ti,ab,kw

#3 MeSH descriptor: [Risk Assessment] explode all trees

#4 (braden near (score* or scale* or tool* or assess*)):ti,ab,kw OR (risk near/2 assess*):ti,ab,kw OR ((assess* or predict*) next (tool* or score* or scale* or instrument* or equipment* or device*)):ti,ab,kw

#5 #1 OR #2

#6 #3 OR #4

#7 #5 AND #6

**Pubmed:**

#1 "Pressure Ulcer"[Mesh]

#2 ((pressure ulcer*[Title/Abstract] OR pressure injur*[Title/Abstract] OR pressure sore*[Title/Abstract] OR pressure damag*[Title/Abstract] OR decubitus ulcer*OR decubitus sore*[Title/Abstract] OR Bedsore*[Title/Abstract] OR bed sore*[Title/Abstract]))

#3 #1 OR #2

#4  "Risk Assessment"[Mesh]

#5 (((risk assess*[Title/Abstract]) OR (braden[Title/Abstract] OR braden score*[Title/Abstract] OR braden scale*[Title/Abstract] OR braden tool*[Title/Abstract] OR braden assess*[Title/Abstract])) OR (assessment tool*[Title/Abstract] OR assessment score*[Title/Abstract] OR assessment scale*[Title/Abstract] OR assessment instrument*[Title/Abstract] OR assessment equipment*[Title/Abstract] OR assessment device*[Title/Abstract])) OR (predict tool*[Title/Abstract] OR predict score*[Title/Abstract] OR predict scale*[Title/Abstract] OR predict instrument*[Title/Abstract] OR predict equipment*[Title/Abstract] OR predict device*[Title/Abstract])

#6   #4 or #5

#7 #6 and #3

**Embase :**

#1 'decubitus'/exp 21030

#2 'pressure ulcer*':ti,ab,kw OR 'pressure injur*':ti,ab,kw OR 'pressure sore*':ti,ab,kw OR 'pressure damage':ti,ab,kw OR 'decubitus ulcer*or decubitus sore*':ti,ab,kw OR bedsore*:ti,ab,kw OR 'bed sore*':ti,ab,kw

#3 #1 OR #2

#4 'risk assessment'/exp

#5 'assessment tool* orassessment score*':ti,ab,kw OR 'assessment scale*':ti,ab,kw OR 'assessment instrument*':ti,ab,kw OR 'assessment equipment*':ti,ab,kw OR 'assessment devices':ti,ab,kw OR 'predict tool*':ti,ab,kw OR 'predict score*':ti,ab,kw OR 'predict scale*':ti,ab,kw OR 'predict instrument*':ti,ab,kw OR 'predict equipment*':ti,ab,kw OR 'predict device*':ti,ab,kw OR 'risk assess*':ti,ab,kw OR braden:ti,ab,kw OR 'braden score*':ti,ab,kw OR 'braden scale*':ti,ab,kw OR 'braden tool*':ti,ab,kw OR 'braden assess*':ti,ab,kw

#6 #4 OR #5

#7 #3AND #6

**CINAHL:**

S1 (MH "Pressure Ulcer+")

S2 (MH "Braden Scale for Predicting Pressure Sore Risk")

S3 S1 AND S2
